# Mitigating effect of tanshinone IIA on ventricular remodeling in rats with pressure overload-induced heart failure ^1^

**DOI:** 10.1590/s0102-865020190080000007

**Published:** 2019-10-14

**Authors:** Xu Li, Daokang Xiang, Yizhu Shu, Xiangjun Zeng, Yonghong Li

**Affiliations:** I Master, Department of Cardiac Surgery , Guizhou Provincial People’s Hospital , Guiyang , China . Design of the study, final approval.; II MD, Department of Cardiac Surgery , Guizhou Provincial People’s Hospital , Guiyang , China . Design of the study, critical revision, final approval.; III MD, Department of Cardiac Surgery , Guizhou Provincial People’s Hospital , Guiyang , China . Conception of the study, final approval.; IV MD, Department of Cardiac Surgery , Guizhou Provincial People’s Hospital , Guiyang , China . Acquisition of data, statistical analyses, final approval.; V MD, Department of Cardiac Surgery , Guizhou Provincial People’s Hospital , Guiyang , China . Manuscript writing, final approval.

**Keywords:** Tanshinone, Ventricular Remodeling, Heart Failure, Myocytes, Cardiac, Apoptosis, Rats

## Abstract

**Purpose:**

To investigate the effect of tanshinone IIA (TIIA) on ventricular remodeling in rats with pressure overload-induced heart failure.

**Methods:**

Pressure overload-induced heart failure model (abdominal aortic coarctation) was established in 40 rats, which were divided into model and 5, 10 and 20 mg/kg TIIA groups. Ten rats receiving laparotomy excepting abdominal aortic coarctation were enrolled in sham-operated group. The 5, 10 and 20 mg/kg TIIA groups were treated with 5, 10 and 20 mg/kg TIIA, respectively, for 8 weeks.

**Results:**

Compared with model group, in 20 mg/kg TIIA group the left ventricular ejection fraction, left ventricular fractional shortening, left ventricular systolic pressure, ±maximum left ventricular pressure rising and dropping rate, and myocardial B-cell lymphoma-2 and cleaved cysteinyl aspartate specific proteinase-3 protein levels were increased, respectively (P<0.05), and the left ventricular end diastolic diameter, left ventricular end systolic diameter, left ventricular end diastolic pressure, heart weight index, left ventricular weight index, serum B-type brain natriuretic peptide, interleukin 6 and C-reactive protein levels and myocardial B-cell lymphoma-2 associated X protein level were decreased, respectively (P<0.05).

**Conclusion:**

TIIA may alleviate ventricular remodeling in rats with pressure overload-induced heart failure heart by reducing inflammatory response and cardiomyocyte apoptosis.

## Introduction

Heart failure refers to the syndrome presented by circulatory system dysfunction due to absolute or relative insufficiency of cardiac output under sufficient venous blood reflux. Heart failure is the last common pathway of various cardiovascular diseases, and has increasingly become a major public health problem threatening the human health ^1^ . The treatment of heart failure is progressing with the deepening of understanding of its pathophysiological mechanism. It has been recognized that the ventricular remodeling is the basic mechanism for the occurrence and development of heart failure ^2^ . Ventricular remodeling refers the changes in cardiac structure, function and phenotype due to a series of complex cellular and molecular mechanisms, including cardiomyocyte hypertrophy, apoptosis, re-expression of embryonic genes and proteins, and changes in extracellular matrix ^3 - 5^ . There are many methods to treat heart failure nowadays. The common used drugs often have some toxic and side effects. In addition, the new treatment methods such as stem cell therapy, pacing therapy, heart transplantation and application of ventricular assist devices are rarely clinically used, expensive, and difficult to be accepted by the common people ^6 - 9^ . *Salvia miltiorrhiza* Bge. is a popular medicinal herb. Modern medicine has proved that *Salvia miltiorrhiza* Bge. can remove the impurities in blood, promote the activity of fibrinolytic enzyme, reduce the blood viscosity, promote thrombolysis, smooth the blood vessels and protect the cardiovascular system ^10^ . Tanshinone IIA (TIIA) is the main active ingredient of *Salvia miltiorrhiza* Bge. TIIA has many biological activities, especially in resisting oxidant stress and reducing inflammatory response aspects ^11 , 12^ . In addition, TIIA has a protective effect on myocardial ischemia ^13^ . Therefore, it is speculated that TIIA may also have protective effects on ventricular remodeling in heart failure. This study investigated the mitigating effect of TIIA on ventricular remodeling in rats with pressure overload-induced heart failure and related mechanisms. The objective was to provide a basis for the development of TIIA-related drugs for heart failure patients.

## Methods

This study was carried out in strict accordance with the recommendations in the Guide for the Care and Use of Laboratory Animals of the National Institutes of Health. The animal use protocol was approved by the Institutional Animal Care and Use Committee of Guizhou Provincial People’s Hospital.

### Establishment of pressure overload-induced heart failure model

The rat model of pressure overload-induced heart failure was established by the abdominal aortic coarctation method. The male SD rats (250-280 g) were anaesthetized using 10% chloral hydrate by intraperitoneal injection. A lower abdominal median incision was made, and the abdominal cavity was opened. The abdominal aorta 0.5 cm above the renal artery branch was bluntly separated. A 8-gauge syringe needle with bluntly grinded tip was placed parallel to the abdominal aorta, and abdominal aorta and syringe needle were ligated using 4 ^#^ surgical thread, followed by slowly withdrawing the syringe needle. Finally, the abdominal cavity was closed with layered suture. In the sham-operated group, the steps were the same as described above, excepting the abdominal aorta ligation. After operation, penicillin (4000 U/kg) was injected intraperitoneally for 3 days to prevent infection. The cardiac function indexes in rats were measured using the Vivid 7 BT08 color Doppler ultrasound diagnostic instrument (GE Medical Company, Shanghai, China). The left ventricular ejection fraction (LVEF) ≤ 60% indicated that the model was successfully established ^14^ . The rats were observed continuously for 4 weeks.

### Animal grouping and treatment

During the molding, 10 rats died. The heart failure model was successfully established in 40 rats, which were randomly divided into model group and 5, 10 and 20 mg/kg TIIA groups, with 10 rats in each group. Ten rats receiving laparotomy excepting abdominal aortic coarctation were enrolled in the sham-operated group. The rats in 5, 10 and 20 mg/kg TIIA groups were intraperitoneally injected with TIIA (98% purity; Xi’an Tianbao Biotechnology Co., Ltd., Xi’an, China), with doses of 5, 10 and 20 mg/kg, respectively. The sham-operated group and model group were intraperitoneally injected with an equal volume of normal saline. The intraperitoneal injection was performed once per day, and was continued for 8 weeks. The feeding, edema, activity and mental status of rats were daily recorded.

### Measurement of cardiac function indexes

At the end of treatment, the cardiac function indexes in rats were measured using the Vivid 7 BT08 color Doppler ultrasound diagnostic instrument. The LVEF, left ventricular end diastolic diameter (LVIDd), left ventricular end systolic diameter (LVIDs) and left ventricular fractional shortening (LVFS) were recorded.

### Detection of hemodynamic indexes

Rats were anesthetized by intraperitoneal injection of pentobarbital sodium, and then were fixed on the operating table. The right common carotid artery was separated. A PE50 artery catheter was inserted into the left ventricle via the right common carotid artery. The left ventricular systolic pressure (LVSP), left ventricular end diastolic pressure (LVEDP), and maximum left ventricular pressure rising and dropping rate ( ± d *p* /d *t*_max_ ) were detected by MP150 biological function experiment system (BIOPAC Systems, Inc, CA, USA). Finally the blood was taken from the common carotid artery, and kept for detection.

### Determination of serum biochemical indexes

Blood of rats was centrifuged at 3000 rpm for 10 min, and the serum was obtained. The serum B-type brain natriuretic peptide (BNP), C-reactive protein (CRP) and interleukin 6 (IL-6) levels were determined using enzyme linked immunosorbent assay. The procedures were in accordance to the instructions of kits (Shanghai Bangyi Biotechnology Co., Ltd., Shanghai, China).

### Detection of heart weight index and left ventricular weight index

After blood collection rats’ hearts were removed. After washing with 0.9% sodium chloride solution, the water on the surface of heart was sucked using clean filter paper. Then the heart was weighed. Then, left ventricle was separated and weighed. The heart weight index (HWI, ratio of heart weight to body weight) and the left ventricle weight index (LVWI, ratio of left ventricle weight to body weight) were calculated.

### Detection of cardiomyocyte apoptosis-related protein expressions in myocardial tissue

The myocardial tissue was homogenized, and the protein was extracted. The expressions levels of cardiomyocyte apoptosis-related proteins including B-cell lymphoma-2 (Bcl-2), Bcl-2 associated X (Bax) and cleaved cysteinyl aspartate specific proteinase-3 (cleaved caspase-3) were determined using western blotting assays ^15^ . The procedures were in accordance to the instructions of kits (Shanghai Yanjie Biotechnology Co., Ltd., Shanghai, China). β-actin was used as the internal reference. The relative expression levels of Bcl-2, Bax and cleaved caspase-3 protein were presented by the ratio of their integral optical density to internal reference, respectively.

### Statistical analysis

All data were expressed as the mean ± SD. The SPSS 22.0 software was performed for statistical analysis. The comparisons among different groups were conducted using one-way analysis of variance (ANOVA) followed by LSD-t test. The value of P<0.05 was considered statistically significant.

## Results

### General conditions of rats

During treatment period, the rats in sham-operated group presented normal hair color, diet, activity and body weight. The rats in model group and 5, 10 and 20 mg/kg TIIA groups showed mental depression, eating loss, decreased body weight and body temperature, decreased food consumption and fur sparse, compared with sham-operated group. These performances were the most obvious in model group and the least apparent in the 20 mg/kg TIIA group.

### Comparison of cardiac function indexes among five groups

**Figure 1:**
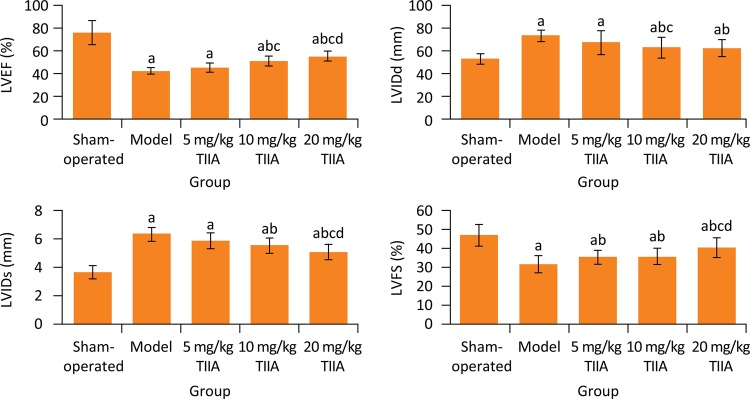
Cardiac function indexes in five groups. a P < 0.05 versus sham-operated group; b P < 0.05 versus model group; c P < 0.05 versus 5 mg/kg TIIA group; d P < 0.05 versus 10 mg/kg TIIA group. TIIA, tanshinone IIA; LVEF, left ventricular ejection fraction; LVIDd, left ventricular end diastolic diameter; LVIDs, left ventricular end systolic diameter; LVFS, left ventricular fractional shortening.

At the end of treatment, the LVEF and LVFS in model groups were significantly lower than those in sham-operated group, respectively (P < 0.05), and the LVIDd and LVIDs in model groups were significantly higher than those in sham-operated group, respectively (P < 0.05). Compared with model group, the LVEF in M-TSN and H-TSN groups and LVFS in 5, 10 and 20 mg/kg TIIA groups were significantly increased, respectively (P < 0.05), and the LVIDd and LVIDs in 10 and 20 mg/kg TIIA groups were significantly decreased, respectively ( Fig. 1 ).

### Comparison of hemodynamic indexes among five groups

**Figure 2:**
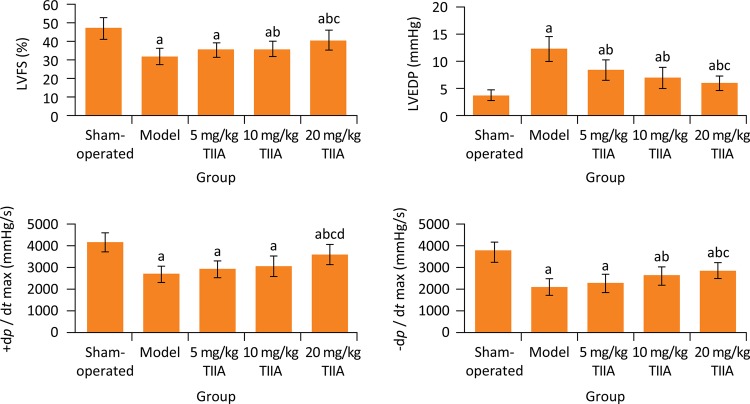
Hemodynamic indexes in five groups. a P < 0.05 versus sham-operated group; b P < 0.05 *vs.* model group; c P < 0.05 *vs.* 5 mg/kg TIIA group; d P < 0.05 *vs.* 10 mg/kg TIIA group. TIIA, tanshinone IIA; LVSP, left ventricular systolic pressure; LVEDP, left ventricular end diastolic pressure; d *p* /d *t* max , maximum left ventricular pressure rising and dropping rate.

After treatment, the LVEDP in model group was significantly higher than sham-operated group (P < 0.05), and the LVSP, +d *p* /d *t*_max_ and -d *p* /d *t*_max_ in model group were significantly lower than sham-operated group, respectively (P < 0.05). Compared with model group, the LVEDP in 5, 10 and 20 mg/kg TIIA groups was significantly decreased, respectively (P < 0.05), the LVSP and -d *p* /d *t*_max_ in 10 and 20 mg/kg TIIA groups and the +d *p* /d *t*_max_ in 20 mg/kg TIIA group were significantly increased, respectively (P < 0.05) ( Fig. 2 ).

### Comparison of HWI and LVWI among five groups

**Figure 3:**
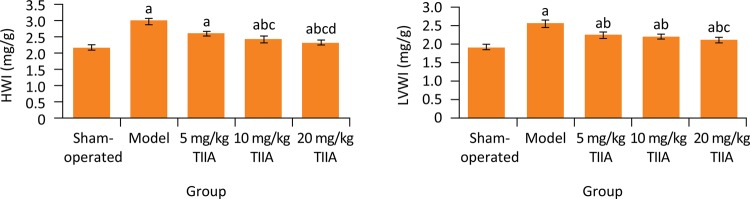
HWI and LVWI in five groups. a P < 0.05 *vs.* sham-operated group; b P < 0.05 *vs.* model group; c P < 0.05 *vs.* 5 mg/kg TIIA group; d P < 0.05 *vs.* 10 mg/kg TIIA group. TIIA, tanshinone IIA; HWI, heart weight index; LVWI, left ventricular weight index.

At the end of treatment, the HWI and LVWI in model group were significantly higher than sham-operated group, respectively (P < 0.05). Compared with model group, the HWI and LVWI in 5, 10 and 20 mg/kg TIIA groups were significantly decreased, respectively (P < 0.05) ( Fig. 3 ).

### Comparison of serum BNP, CRP and IL-6 levels among five groups

**Figure 4:**
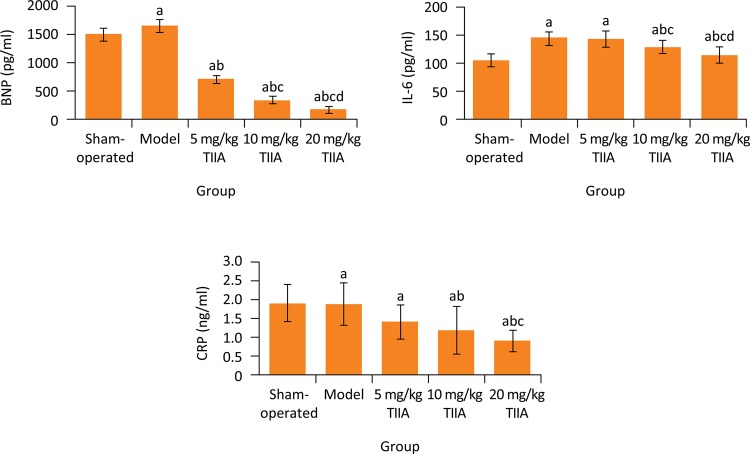
BNP, IL-6 and CRP levels in five groups. a P < 0.05 *vs.* sham-operated group; b P < 0.05 *vs.* model group; c P < 0.05 *vs.* 5 mg/kg TIIA group; d P < 0.05 *vs.* 10 mg/kg TIIA group. TIIA, tanshinone IIA; BNP, B-type brain natriuretic peptide; IL-6, interleukin 6; CRP, C-reactive protein.

Blood biochemistry showed that, the serum BNP, CRP and IL-6 levels in model group were significantly higher than in sham-operated group, respectively (P < 0.05). Compared with model group, the serum BNP level in 5, 10 and 20 mg/kg TIIA groups and serum IL-6 and CRP levels in 10 and 20 mg/kg TIIA groups were significantly decreased, respectively (P < 0.05) ( Fig. 4 ).

### Comparison of myocardial Bcl-2, Bax and cleaved caspase-3 protein expression levels among five groups

**Figure 5:**
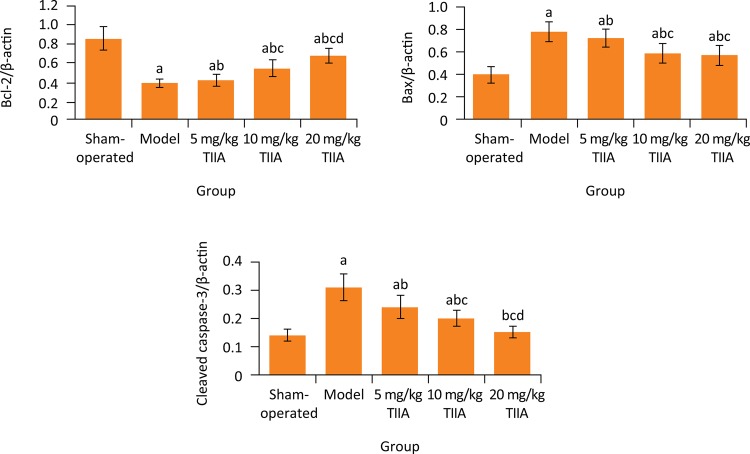
Relative expression levels of myocardial Bcl-2, Bax and cleaved caspase-3 protein in five groups (ratio to β-actin). a P < 0.05 *vs.* sham-operated group; b P < 0.05 *vs.* model group; c P < 0.05 *vs.* 5 mg/kg TIIA group; d P < 0.05 *vs.* 10 mg/kg TIIA group. TIIA, tanshinone IIA; Bcl-2, B-cell lymphoma-2; Bax, Bcl-2 associated X; Cleaved caspase-3, cleaved cysteinyl aspartate specific proteinase-3.

As found in western blotting, compared with sham-operated group, in model group the myocardial Bcl-2 and cleaved caspase-3 protein expression levels were significantly decreased, respectively (P < 0.05), and the myocardial Bax protein expression level was significantly increased (P < 0.05). Compared with model group, the Bcl-2 protein expression level in 10 and 20 mg/kg TIIA groups and cleaved caspase-3 protein expression level in 5, 10 and 20 mg/kg TIIA groups were significantly increased, respectively (P < 0.05), and the Bax protein expression level in 5, 10 and 20 mg/kg TIIA groups was significantly decreased, respectively (P < 0.05) ( Fig. 5 ).

## Discussion

In this study, the pressure overload induced-heart failure model of rats was successfully established by abdominal aorta constriction method, and the protective effect of TIIA on ventricular remodeling in this model was investigated. Results showed that, at the end of treatment, compared with model group, in TIIA group with certain dose the LVEF and LVFS were significantly increased, and the LVIDd and LVIDs were significantly decreased; the LVEDP was significantly decreased, and the LVSP, -d *p* /d *t*_max_ and +d *p* /d *t*_ma_ were significantly increased; the HWI and LVWI were significantly decreased. This suggests that TIIA can alleviate the ventricular remodeling in rats with pressure overload induced-heart failure. This is similar with a previous study ^16^ .

BNP is a 17-ring polypeptide neuroendocrine hormone composed of 32 amino acids. It is mainly synthesized and secreted by ventricular myocytes, and is widely distributed in heart, lung, brain, spinal cord and other tissues. The content of BNP in the heart is the highest. The production of BNP is easily induced by stress, and is sensitive to the changes in ventricular volume. When the ventricular volume and pressure load increase, abnormal hemodynamics occur, and the ventricular wall tension increases, so the amount of BNP secreted by ventricular myocytes to peripheral blood increases ^17^ . BNP can inhibit renin-angiotensin-aldosterone system, and has diuretic, sodium diuretic and vasodilator effects ^18^ . BNP is not interfered by other factors, and is the most sensitive and specific indicator to reflect the changes of ventricular function ^19^ . Therefore, the level of blood BNP can fully reflect the heart function of patients. The most common serious complications of heart failure are the left ventricular hypertrophy and severe function impairment. Studies have shown that there is a positive correlation between blood BNP level and cardiovascular events in heart failure patients ^20 , 21^ . Results of this study showed that, compared with model group, the serum BNP level in three TIIA treatment groups was significantly decreased. This further confirms the protective effect of TIIA on ventricular remodeling in rats.

IL-6 is a multifunctional cytokine that regulates immune response and promotes inflammatory response. A previous study has shown that in heart failure the sympathetic nerve excitation system and renin-angiotensin-aldosterone system are activated, leading to increase of norepinephrine and increased expression of IL-6 in vascular endothelial cells and smooth muscle cells ^22^ . IL-6 mainly exerts the cytotoxic effects on cardiomyocyte. It directly injures the cardiomyocytes, promotes cardiomyocyte hypertrophy, aggravates the cardiac ventricular remodeling, inhibits excitation-contraction coupling of cardiomyocytes, thus producing negative myodynamic effects and participating in the occurrence and development of heart failure ^23^ . Results of this study showed that, compared with model group, the serum IL-6 level in three TIIA treatment groups was significantly decreased. This indicates that, TIIA can reduce the IL-6 due to heart failure, thus alleviating ventricular remodeling.

CRP is one of the most important factors in body, and it is also one of the main factors leading to atherosclerotic diseases. Under physiological conditions, CRP can activate the complement system and remove the pathological substances, and is an important defense system in the body. However, excessive CRP can reduce the endothelial cell function and affect the coagulation and fibrinolysis, seriously increasing the risk of cardiovascular diseases ^24^ . Clinical study has shown that in patients with coronary heart disease, the CRP level is significantly increased, and is about twice of that of healthy people ^25^ . In patients with acute myocardial infarction, the CRP level is obviously higher than that of healthy people ^26^ . Other study has shown that the CRP level is significantly increased in patients with heart failure, and the increase degree is positively correlated with the severity and prognosis of heart failure. Therefore, CRP can be used as an independent index to judge and predict the severity and prognosis of heart failure ^27^ . In the present study, compared with model group, the serum CRP level in three TIIA treatment groups was significantly decreased. This suggests that TIIA can reduce the excessive CRP due to heart failure, thus exerting a protective effect on ventricular remodeling.

Apoptosis is regulated by intracellular apoptotic regulatory proteins, which are divided into apoptotic proteins and anti-apoptotic proteins. Apoptosis is the result of the imbalance between these two antagonistic proteins. Bcl-2 is the main apoptotic suppressor gene. When cells are damaged, their proliferation is inhibited or apoptosis occurs, the expression of Bcl-2 protein decreases, and the associated apoptotic effector Caspase-3 is initiated ^28^ . Caspase-3 is the initiator and executor of apoptosis. It is a common downstream effector of apoptotic pathways mediated by multiple death receptors. Caspase-3 is also an apoptotic suppressor gene ^29^ . Bax is an apoptosis-promotion gene. It cannot only inhibit the apoptosis inhibition effect of Bcl-2, but also directly promote apoptosis of cells ^30^ . In this study, the western blotting showed that, compared with sham-operated group, in model group the myocardial Bcl-2 and cleaved caspase-3 protein expression levels were significantly decreased, and the myocardial Bax protein expression level was significantly increased. Compared with model group, the Bcl-2 and cleaved caspase-3 protein expression levels in 10 and 20 mg/kg TIIA groups were significantly increased, and the Bax protein expression level was significantly decreased. This indicates that cardiomyocyte apoptosis is involved in the ventricular remodeling in rats with pressure overload induced-heart failure, and TIIA can inhibit cardiomyocyte apoptosis.

In summary, TIIA can mitigate the ventricular remodeling and cardiac function in rats with pressure overload-induced heart failure. The underling mechanism may be correlated with it reducing inflammatory response and cardiomyocyte apoptosis in the body. TIIA has a therapeutic potential for clinical treatment of pressure overload-induced heart failure. This study has provided a basis for the development of TIIA-related drugs for heart failure patients. This study still has some limitations. Firstly, other mechanisms related to the therapeutic effect of TIIA on heart failure have not been investigated. Secondly, the sample size of this study is relatively small, which may affect the results. These issues should be considered in further studies for obtaining more convincing outcomes.

## Conclusion

TIIA may alleviate ventricular remodeling in rats with pressure overload-induced heart failure heart by reducing inflammatory response and cardiomyocyte apoptosis.

## Footnotes

1 Research performed at Central Laboratory, Guizhou People’s Hospital, China.
